# Date Fruit Extract Is a Neuroprotective Agent in Diabetic Peripheral Neuropathy in Streptozotocin-Induced Diabetic Rats: A Multimodal Analysis

**DOI:** 10.1155/2011/976948

**Published:** 2011-12-01

**Authors:** Nasser Zangiabadi, Majid Asadi-Shekaari, Vahid Sheibani, Mandana Jafari, Mohammad Shabani, Ali Reza Asadi, Hale Tajadini, Morteza Jarahi

**Affiliations:** ^1^Department of Basic Neuroscience, Neuroscience Research Center, Kerman Medical University, Kerman 76198-13159, Iran; ^2^Afzal Research Center, Kerman, Iran

## Abstract

*Background*. To study the effects of an aqueous extract of date fruit (*Phoenix dactylifera* L. Arecaceae) diet on diabetic polyneuropathy (DPN) in streptozotocin- (STZ-) induced diabetic rats. *Methods*. The effects of a date fruit extract (DFE) diet on diabetic neuropathy in STZ-induced diabetic rats were evaluated and compared with a nondiabetic control group, diabetic control group (sham), and vehicle group with respect to the following parameters: open field behavioral test, motor nerve conduction velocity (MNCV), and morphological observations. *Results*. In the model of STZ-induced of diabetic neuropathy, chronic treatment for 6 weeks with DFE counteracted the impairment of the explorative activity of the rats in an open field behavioral test and of the conduction velocity of the sciatic nerve (MNCV). In addition, pretreatment with DFE significantly reversed each nerve diameter reduction in diabetic rats. *Conclusion*. DFE treatment shows efficacy for preventing diabetic deterioration and for improving pathological parameters of diabetic neuropathy in rats, as compared with control groups.

## 1. Introduction

Diabetes mellitus is a chronic metabolic disorder that leads to long-term complications affecting some tissues such as heart, kidney, retina, and peripheral nerves. Peripheral neuropathy is one of the most frequent and potentially severe complications of diabetes, leading to pain, skin ulcers, muscle weakness, loss of independence, and overall impairment of the patient's quality of life. Diabetic neuropathy is a multifaceted complication of both type I and II diabetes, with an estimated prevalence between 50 and 90% depending on the duration of diabetes and involves a spectrum of functional and structural changes in peripheral nerves [1]. Early disorders of nerve function include slowing in nerve conduction velocity followed by axonal degeneration, paranodal demyelination and loss of myelinated fibers [2]. Experimental DPN shares a number of features with human DPN, such as the structural, functional, and biochemical alterations [3, 4].

At the pathological level, the decrease of intraepidermal nerve fiber density in diabetics with symptoms of neuropathy correlates with electrophysiological and psychophysical Abnormalities [5–7]. Several interactive pathogenetic mechanisms of DPN have been identified in both human and murine models and persistent hyperglycaemia has been regarded as a primary risk factor for neuropathy [8].

 Long-term hyperglycaemia leads to subsequent enhanced oxidative stress, increased aldose reductase activity, accumulation of advanced glycation endproducts, and decreased Na^+^, K^+^-ATPase activity [9–12]. Diabetic neuropathy is also thought to develop as a result of damage caused by oxidative stress and other vascular risk factors [13, 14]. As a result, it could induce progressive damage to the peripheral sensory, motor, and autonomic nervous systems [15]. Current treatment of diabetic neuropathy relies on the control of glycemic, oxidative stress, and neural and vascular risk factors [13, 14], but this does not fully prevent its occurrence or progression. To date, except for rigorous glycaemic control, there are few means to affect or slow the natural progression of DPN owing to limitations of the current inadequate drug therapy [16]. Treatment of diabetic neuropathy is therefore a major goal but, despite multiple attempts, no satisfactory management is yet available. Consequently, the search for substances to protect the nervous system from the degenerative effects of diabetes has high priority in biomedical research.

DFEs might be interesting since they have been recently identified as promising neuroprotective agents in several models of neurodegeneration [17]. Recent findings suggest that antioxidant agents might exert neuroprotective effects and may be promising in therapy. Several dietary supplements have been reported to have strong antioxidant effects and reduce neurological deficits in aged animals [18]. One of the dietary supplements is date fruit which has great importance from nutritional and economic points of view and it is composed of a fleshy pericarp and seed [19]. The importance of the date in human nutrition comes from its rich composition of carbohydrates, salts and minerals, dietary fibers, vitamins, fatty acids, amino acids, and protein. In many ways, date may be considered as an almost ideal food [20]. In addition, the date palm fruit possesses many useful properties such as antioxidant and antimutagenic [21], antibacterial [22], antifungal [23], antitumoral [24], and gastrprotective [25] properties. Meanwhile, date fruit has various neuroprotective agents such as melatonin [26] and polyphenolic compounds [19].

According to recent studies, by Asadi-Shekaari et al. and Panahi et al., DFE significantly inhibited neuronal damage induced by cerebral ischemia [17, 27]. Also they resulted that the effectiveness of DFE in focal cerebral ischemia is most probably is due to its antioxidant property [17]. 

We have assessed whether the use of DFE has neuroprotective effects against STZ-induced diabetic neuropathy at the neurophysiological, behavioral, and neuropathological levels. STZ, which induces type 1 diabetes mellitus when delivered to rats, is a useful etiological model for studying complications caused by diabetic hyperglycemia [28]. Reduced nerve conduction velocity was found by numerous authors in STZ-diabetic rats [29, 30]. The reduction of Na^+^, K^+^-ATPase activity, together with the decrease in NCV, is the hallmark of diabetic neuropathy [31]. 

 We were interested in investigating the effects of DFE-enriched diets on the development of diabetic neuropathy in STZ-induced diabetes in rats. We decided to investigate the effect of DFE-enriched diets specifically on MNCV, behavioral activities, and pathology in STZ-induced diabetes in rats. 

## 2. Materials and Methods

### 2.1. Drugs and Reagents

To prepare DFE, fresh date fruit was prepared from Bam town orchards, Kerman province (South East of Iran). The kernel was removed and 100 gr of date was immersed in 1000 mL/distilled water for 48 hours. It was then mixed completely in a mixer and then the mixture was centrifuged at 4000 rpm at 4°C for 20 minutes. After sedimentation, the supernatant part was used for gavages. Streptozotocin was purchased from Sigma-Aldrich. Blood glucose kits were purchased from the Roche Company, Germany.

### 2.2. Animals and Experimental Protocols

The care of laboratory animals followed the guiding principles for care and use of laboratory animals of the Neuroscience Research Center of Kerman Medical University and the study protocol was approved by the animal ethics committee of this institution (Code: EC/KNRC/88-15). This study was carried out on male rats of Wistar breed weighing 250 ± 20 g. During the study period, experimental animals were given standard pellet diet and water ad libitium, kept in the laboratory animal house under specific pathogen-free (SPF) and constant temperature (25 ± 1°C) conditions and a 12 h light-dark cycle (lights on at 06:30 h). Special care was taken to minimise animal suffering and to reduce the number of animals used to the minimum required for statistical accuracy. Animals were acclimatised to laboratory conditions before the tests. All experiments were carried out between 09:00–13:00 h.

Diabetes was induced by intraperitoneal injection of 45 mg/kg freshly prepared STZ dissolved in 0.1 mol/L Citrate buffer (pH 4.4). At the end of the first week after STZ injection, blood sugar measurements were taken for diagnosing diabetes induction using tail's vein blood (by ACCU-CHEK-ACTIVE kit made by Roche, Germany). Rats with fasting blood glucose levels over 200 mg/dL were used in the experiments [32]. The animals were divided into four groups: the first group consisted of nondiabetic animals. Age- and weight-matched male Wistar rats that had not been made diabetic were used as controls. The second, third, and fourth groups consisted of diabetic animals. The second group was as diabetic control group (sham). The third and fourth groups were pretreated with DFE and vehicle (4 mL/kg/day, orally). Each group consists of 8 rats. In this study, to prevent diabetic neuropathy, DFE (4 mL/kg, daily as gavage) was used for a period of six weeks, after rats were confirmed diabetic. Similarly to the DFE group, the vehicle group also received distilled water (4 mL/kg, daily as gavage) from the end of the first week for a period of six weeks. 

### 2.3. Physiological Measurements

Six weeks after starting intervention, the horizontal and vertical activities of the male rats from each group were measured in an open field with a computerized Ethovision software (version 3.1), a video tracking system for automation of behavioral experiments (Noldus Information Technology, The Netherlands). Black wooden box (45 × 45 cm, height: 50 cm) was used. Rats were placed individually in the center of the test box. After that, the following behavioral parameters: total distance moved (TDM, cm), total duration mobility and immobility, and frequency of rearing (as a measure of vertical activity) and grooming (rubbing the body with paws or mouth and rubbing the head with paws) in the open field were analyzed for 5 min [33]. Mean values (±S.E.M.) were calculated for each and expressed as percent change versus control.

### 2.4. Nerve-Conduction Velocity Measurements

Slowing of sensory nerve conduction velocity (SNCV) and motor nerve conduction velocity (MNCV) was shown to develop within the first month of the onset of hyperglycemia in STZ-diabetic rats [34–36]. Six weeks after the onset of hyperglycemia the animals were anesthetised with 50/20 mg kg—ketamine/xylazine i.p. injection to prevent discomfort and then backs of rats were shaved. During the study, rectal temperature was maintained at 37°C to ease animal stress from anesthetic. In a temperature-controlled environment (25 ±  1°C), small incisions were made in the right sciatic notch and ankle. Sciatic-tibial motor MNCV was measured by stimulating proximally at the sciatic notch and distally at the knee via bipolar needle electrodes (PowerLab/ML856; AD Instruments, Sydney, NSW, Australia; frequency 20 Hz, duration 0.1 ms, amplitude 1.5 V, sampling 20 k/s) [37–39]. After single stimulus the compound muscle action potential was recorded from the first interosseous muscle of the hind-paw by unipolar pin electrodes. The recording was a typical biphasic response with an initial M-wave, which is a direct motor response due to stimulation of motor fibers. The MNCV was calculated as the ratio of the distance (in mm) between both sites of stimulation divided by the difference between proximal and distal latencies measured in ms [37, 38]. Latency is measured from initial onset to maximum negative peak.

### 2.5. Morphological Observations and Analysis of Sciatic Nerve

In order to study morphological alterations resulting from diabetic neuropathy, sciatic nerve was isolated and divided into 2 mm segments. They were fixed immediately with glutaraldehyde solution 2.5% in 0.1 M Phosphate Buffer Solution (PBS) for 24 hr. The specimens were washed with PBS for 3 times and post fixed for 2 hr in 1% tetroxide osmium and dehydrated in graded concentration of ethanol and finally embedded in Epon 812 resin. Semithin sections (300 nm) were stained with 1% toluidin blue and examined by light microscopy. Ten fields of transverse sections were morphometrically analyzed by computerized image analysis system (Motic Images China e-kup Co.,Ltd). MFD, AD and MSD were measured for each section. For ultrastructural study, ultrathin sections (70 nm) were stained with 1% uranyl acetae and 2% lead citrate before viewing in Philips EM300 (Philips, Eindhoven, Netherlands).

### 2.6. Statistical Analysis

The parametric data obtained by the experiments have been analyzed by using SPSS version 17.0. The quantitative data are expressed as the mean ± SEM. Multiple comparisons were performed among experimental groups by one-way analysis of variance (ANOVA) followed by Tukey-Kramer post hoc test.   *P* < 0.05 was considered statistically significant.

## 3. Results

### 3.1. Metabolic Characteristics

In the diabetic groups, the plasma glucose concentrations at the end of the first week were 296.19% greater than those of control group (*P* < 0.0001). All diabetic rats showed high blood glucose and marked impairment of growth at the end of the 7th week after STZ injection. As shown in Table 1, body weight of 7th-week diabetic rats was significantly lower than normal control rats. Treatment with DFE (six weeks) did not significantly affect the blood glucose levels in diabetic rats but there was a significantly difference in weight gain compared to the sham group (*P* < 0.01).

### 3.2. Effects of DFE on Open Field Test

**Figure 1:**
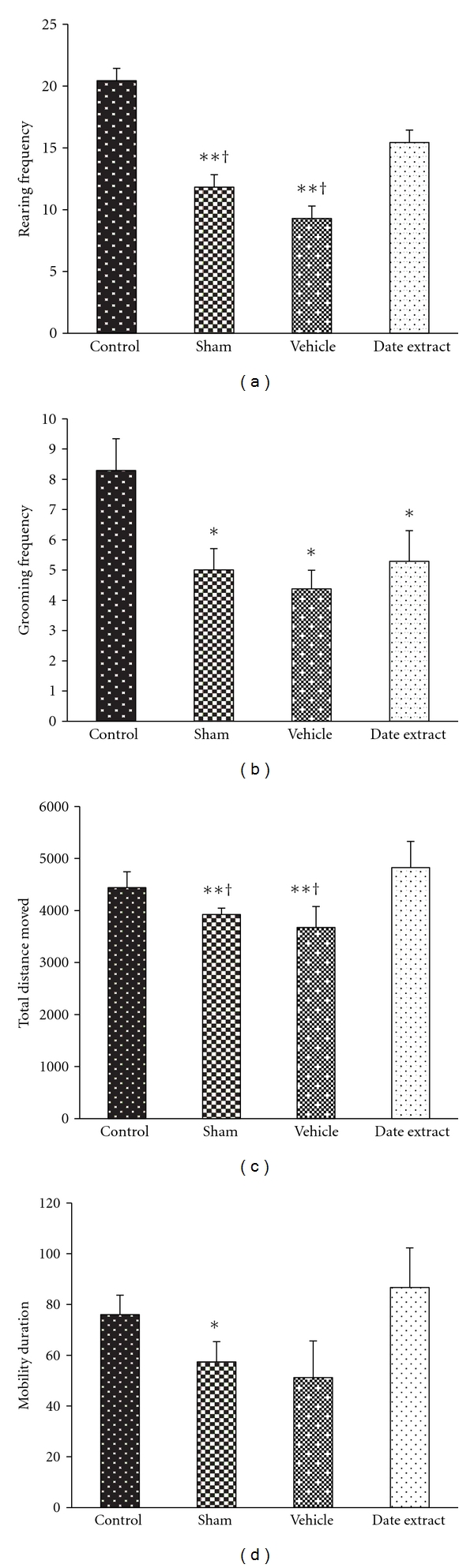
Effect of Date fruit extract on explorative behavior of rats in open field test. (a) Rearing frequency: **P < 0.01 compared with the control group, ^†^ *P* < 0.05, compared with the DE group. (b) Grooming frequency: *P < 0.05 compared with the control group and (c) TDM: **P < 0.01 compared with the control group, ^†^ *P* < 0.05 compared with the DE group. (d) Mobility duration: *P < 0.05 compared with the control group. Data are the Mean ± SEM.

In the assessment of diabetic neuropathy on the explorative activity of rats in an open field behavioral test, TDM, mobility, rearing and grooming frequency were significantly decreased in sham group compared with the control group, but there were no significant differences between control, sham, and DFE groups in time spent either in the centre (the most anxiogenic area) or in the perimeter of the open field. Analysis of the TDM and mobility showed that DFE group rats travelled significantly more than rats in the sham group (*P* < 0.05) in the central and peripheral arenas. Rearing frequency significantly increased in the DFE group compared with the sham group (*P* < 0.05), while grooming frequency decreased significantly in all of diabetic groups compared with control group (*P* < 0.05); see Figure 1.

### 3.3. Effects of DFE on MNCV

**Figure 2:**
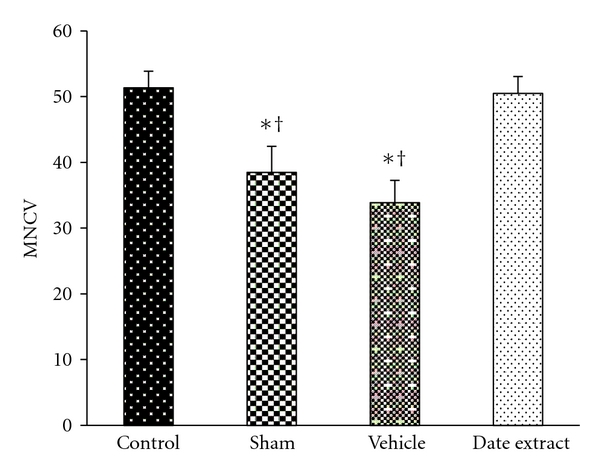
Effect of Date fruit extract on motor nerve conductive velocity (MNCV) in rats. Data are the Mean ± SEM. *P < 0.05 compared with the control group; ^†^ *P* < 0.05, compared with the DE group.

Seven weeks after STZ injection, the sciatic MNCV of rats in the sham group (38.50 ± 2.06 m/s) compared with that in the nondiabetic control group (51.36 ± 3.5 m/s) was significantly slower by approximately 133.4%. The MNCV in diabetic rats treated with DFE showed a statistically significant increase compared with that of diabetic rats in the sham group (*P* < 0.05) (Figure 2). The sciatic MNCV for rats in the DFE group did not differ from the control group.

### 3.4. Effects of DFE on Morphological Changes of Myelinated Nerves

**Figure 3:**
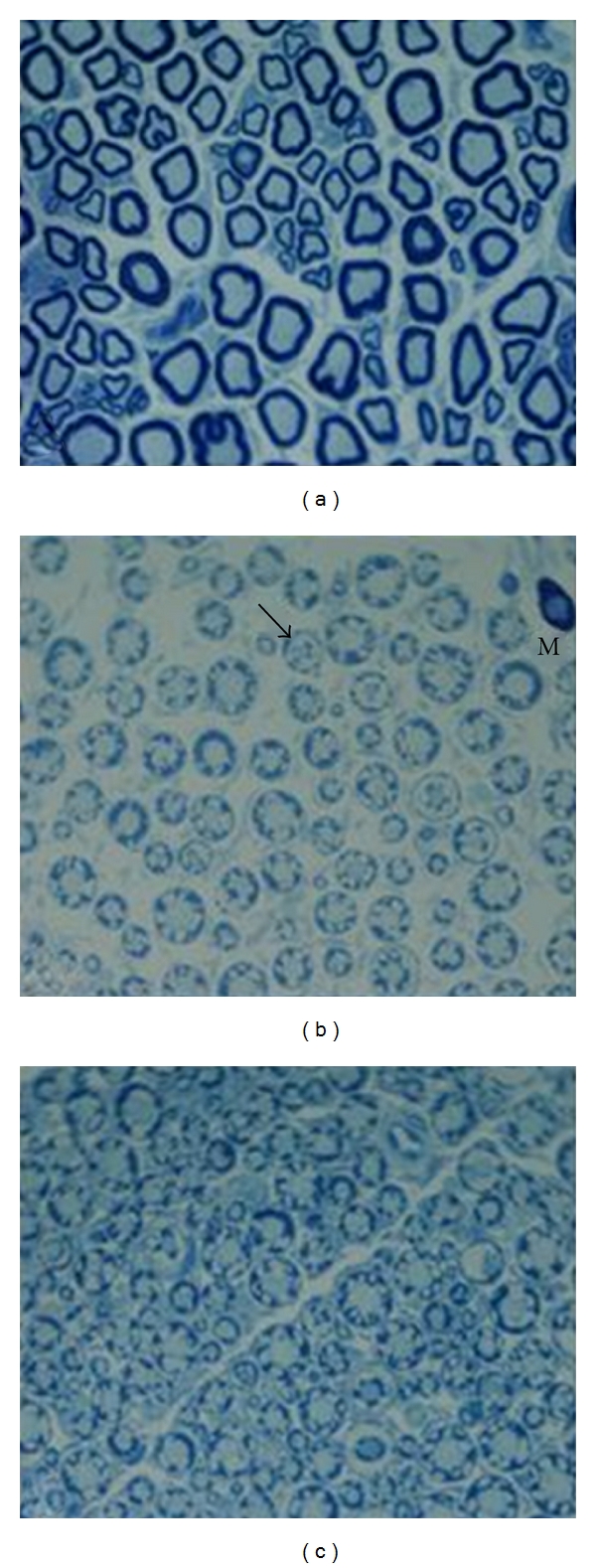
Light micrograph of transverse semithin sections of rat sciatic nerves. (a) Control group: myelinated nerve fibers are in normal structure and morphology. (b) Sham group: nerve fibers show some abnormalitities such as myelin splitting (black arrow), mast cell infiltration (M), and edema. (c) The DFE-treated group; the proportion of nerve fibers with abnormalities was reduced ×1000 (Toluidin blue staining).

Light microscopy study of sciatic nerves sections showed the normal structure and morphology of myelinated fibers in the control group. In the sham and vehicle groups, some abnormalities including increased numbers of mast cells, edema, and myelin sheath splitting were seen (Figure 3). Increasing in number of abnormal myelinated fibers was observed in Sham and vehicle groups. Pretreatment of rats with DFE prevented all of these abnormalities to a high extent. According to the data, MFD and AD of vehicle and Sham groups were decreased in comparison to control group. Pretreatment with DFE for six weeks significantly reversed each diameter reduction in diabetic rats (Table 2).

### 3.5. Electron Microscopy

**Figure 4:**
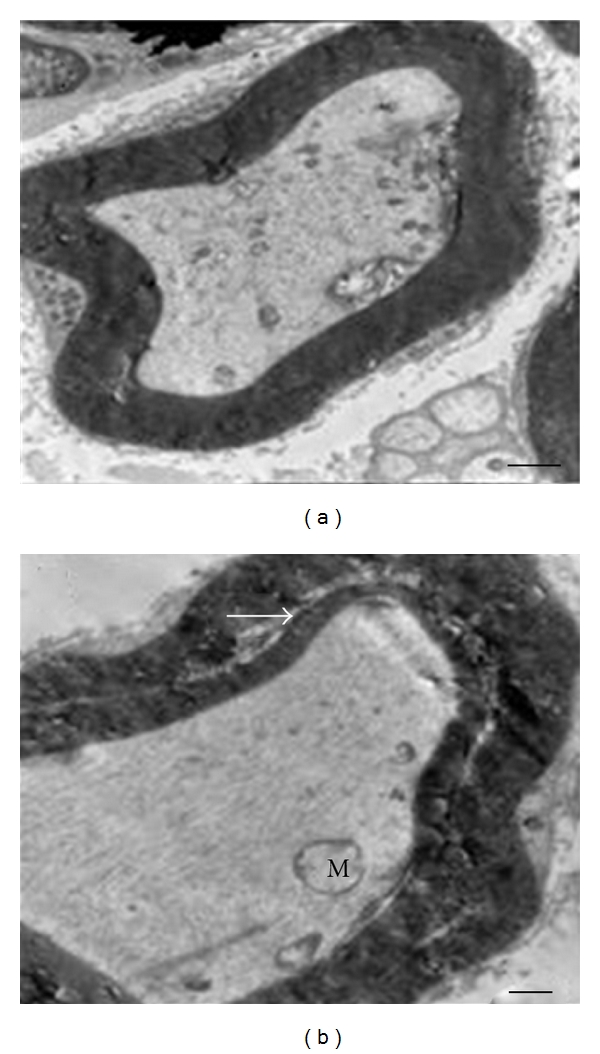
Electron micrograph of rat sciatic nerves. (a) Control group: myelinated fiber with normal structure and morphology. Axon is in normal condition too. (b) Sham group: Myelinated fiber shows myelin splitting (white arrow). Loosening of myelin lamellae may account for paler staining in the outer myelin sheath. Axonal degeneration was revealed with enlarged mitochondria (M) and neurofilament disintegration ((a) ×8900, (b) ×11500).

Ultrastructural evaluation of sciatic nerves confirmed the light microscopy findings. In addition, some evidences of axonal degeneration such as mitochondrial swelling and disintegration of neurofilaments were observed under transmission electron microscope (Figure 4) but degenerative, reactive, and proliferative of Schwann cells, were not observed in any groups.

## 4. Discussion

In the present study, we evaluated the neuroprotective effects of a DFE diet on the development of experimental neuropathy. According to our data, DFE (4 mL/kg/day, orally) has a beneficial effect in the prevention of experimental neuropathy in male rats. In fact, we found a positive effect of DFE on electrophysiological marker of diabetic neuropathy (MNCV) and open field behavioural test. Furthermore, we showed that DFE partially prevented the deficit in the pathological morphology of myelinated sciatic nerve fibers in diabetic rats.

Patterns of diabetes-related peripheral nerve abnormalities can be categorised as distal symmetric sensory motor neuropathy, autonomic neuropathy, and focal asymmetric neuropathy. The most common pattern of injury is the symmetric neuropathy that involves distal sensory and motor nerves; small sensory fiber neuropathy is often prominently affected in the form of peripheral neuropathy of diabetic mellitus. With increased duration of the disease, diabetic subjects developed decreased sensations in the distal extremities that will result later in the loss of pain sensation and ulcers. Treatment of diabetic neuropathy is a major goal, but despite multiple attempts, no satisfactory management is yet available. Moreover, clinical data have shown that diabetic patients suffering peripheral neuropathy manifest electrophysiological conduction abnormalities [40]. Progressive slowing of MNCV is an important characteristic related to DPN. Our experiments showed that diabetic neuropathy was induced by STZ at six weeks, as evidenced by slowing of NCV (Figure 2), in agreement with the literature [41]. We observed a 25% deficit in MNCV in diabetic rats as compared to nondiabetic rats. These results are in accordance with other reports [37, 42–45]. In the present experiments, diabetic rats treated with DFE exhibited amelioration of the changes in MNCV.

Abnormal fibers undergoing axonal degenerative changes and myelin breakdown are reported to appear in the sciatic nerve of STZ-induced diabetic rats [46]. We saw pathological changes consisting mainly of axonal degeneration in the sciatic nerves in the diabetic rats after seven weeks of STZ injection (Table 2). In our current study, the efficacy of the DFE diet was also confirmed by morphometric analysis showing that axonopathy predominates over myelinopathy in STZ-diabetic rats. We measured histomorphometric parameters MMFD, AD, and MSD of sciatic nerves specifically. These morphological indices are corrected by the DFE diet, giving an axonal diameter distribution similar to that for the nondiabetic animals.

Diabetes-induced slowing of conduction of large-myelinated fibers (LMFs) was accompanied by a decrease in the proportion of the largest fibers present in the sciatic nerve. There is no marked fiber loss in short-term diabetic rats, and the reduction in the proportion of large fibers was accompanied by a parallel increase in the proportion of medium-sized fibers, suggesting either delayed radial growth of axons or atrophy of large axons. As axonal caliber is a major determinant of conduction velocity, the capacity of DFE to restore or maintain the proportion of large myelinated fibers in the sciatic nerve of diabetic rats reveals a potential mechanism that could explain the effect of DFE on conduction. Physiopathology of diabetic neuropathy includes activation of the polyol pathway, increases in oxidative stress and advanced glycosylation end products, perturbation in neurotrophism, and abnormalities in essential fatty acids metabolism [47]. Moreover, previous studies have shown that free radicals induce oxidative stress under diabetic conditions to hyperglycaemia [37, 44, 48, 49].

Oxidative stress causes vascular impairment leading to decrease in nerve blood flow, resulting in endoneurial hypoxia and impaired neural function which may cause MNCV slowing [37, 50]. On the other hand, according to earlier investigations, LMFs preeminently determine the compound MNCV [51]. Therefore, the decrease in MNCV may be due to a decrease in LMFs or the arrested developmental process of MFs. Moreover, the decrease in MNCV could result from alterations in Na^+^, K^+^-ATPase activity, the membrane environment, and histological damage that particularly cause the loss of MLFs [52]. Treatment of STZ-induced diabetic rats with DFE improved MNCV, which could be due to preserved fibres and axon diameter.

Our findings, in accordance with earlier studies, indicate that treatment with STZ increases morphological alteration in the MFs of the sciatic nerve. The most abundant myelin abnormality observed in our study was myelin infolding in the axoplasm and fiber irregular shapes (Figure 3). Its frequency is increased in aging and different peripheral neuropathies including peripheral diabetic neuropathy [53, 54]. In the present study some of the significant effects of treatment with DFE are the reduction in the frequency of axons with myelin abnormalities and loss of the proportion of fibers with irregular shapes induced by STZ treatment. In agreement with earlier findings, our present results indicate that a significant decrease in the MMFD and MSD was also detected in STZ-induced diabetic rats [54]. Treatment of STZ rats with DFE is able to counteract the decrease in MMFD and MSD in the sciatic nerve. 

Electrophysiological and morphologic observations have been confirmed by the explorative behavior of animals observed in the open field test. In this test, TDM, rearing, and mobility are signs of alterations in the animal's spontaneous movements and explorative behaviour that showed a significant decrease in diabetes due to diabetic neuropathy. In agreement with our results, significantly decreased locomotor and exploratory activity (number of crossed fields, rearings, and bar approaches) have been reported in STZ-diabetic rats [55].

According to the results of the open field test, in the group that received DFE, as opposed to the sham group, TDM, mobility, and rearing showed a significant increase and became close to the control group. Therefore, it can be concluded that the neuroprotective effect of DFE in the prevention of diabetic neuropathy and consequently the prevention of defects in explorative behaviour due to diabetes is significant. 

As mentioned above, the pathology of diabetic neuropathy also involves polyol pathway flux, oxidative stress, accumulation of advanced glycation endproducts, and microvascular injuries. Oxidative stress is the cause as well as sequel of almost all major pathophysiological pathways in diabetic neuropathy. Growing attention has been paid to the pathogenic roles of oxidative stress in diabetic neurological complications. Reactive oxygen species (ROSs) are generated by nonenzymatic protein glycation through a complex series of chemical and cellular intermediates [56]. 

 Recent studies showed that the Iranian dates are strong radical scavengers and can be considered as a good source of natural antioxidants for medicinal and commercial uses [57]. This creates the impression that different altered mechanisms may be involved that the DFE could be having an antioxidant and neuroprotective effect [17, 21, 58]. Some of the established neuroprotective constitituents of DFE are as follows: melatonin, a potent antioxidant and free radical scavenger [59]; Vitamin C, an accepted antioxidant agent [60]; phenolic compounds which are contained in cinnamic acids and distinctive flavonoids with strong antioxidant effects [19]; magnesium, an antagonist of NMDA receptors [61]; Vitamin B_3_, a water-soluble vitamin with neuroprotective properties [62]; manganese, a free radical scavenger; and selenium, an antioxidant factor with synergic effects with vitamin C [63]. Therefore, the possible mechanism that maybe responsible for DFE protection against neuropathy is inhibition of oxidative damage due to ROS as indicated by studies in STZ-diabetic rats treated with DFE. However, it seems that the neuroprotective effect of DFE cannot be mediated by an improvement in the symptoms of diabetes because there is no change in the hyperglycaemic state of diabetic rats.

 Although the animals in the nondiabetic control group consumed the diet as much as diabetic groups, the difference of weight gain among them was significant (Table 1). As the total energy available from protein, fat, and carbohydrate did not differ between control and sham groups, the same intake of diet suggests that STZ-diabetic rats cannot sufficiently utilize the energy available from their diet. As in the other diabetic animals, the DFE-treated diabetic animals lost body weight and remained hyperglycaemic. Although the body weight gain of the DFE group was better than other diabetic groups, we can say that the effects of DFE treatment on nerve function in diabetic rats were not due to a global amelioration of the diabetic condition. Nevertheless, the protective mechanism and connected factors are not known exactly but the synergic effect of different antioxidative components of DFE may give a reason for its neuroprotective mechanism. Supplementary investigation should be designed to make the exact mechanism clear.

To sum up, our study shows that treatment with DFE can decrease behavioral, neurophysiologic and pathological alterations induced by diabetes in the peripheral nerves of rats and that an antioxidant-related mechanism contributed to the improvement of diabetic neuropathy.

## 5. Conclusion

We found that DFE-diet intake in STZ-induced diabetic rats provided protection against deterioration of the peripheral nerve. We conclude that antioxidative property is one of the major profiles that is implicated in neuron protection. Thus our findings suggest that this compound may be considered as a potential preventative approach for peripheral diabetic neuropathy.

## Figures and Tables

**Table 1:** Body weight and blood glucose levels of all groups.

Animal group	Body weight (g)		Blood glucose (mg dL^−1^)
	Before STZ injection	End experiment	Before sacrifice
Control (8)	265 ± 4.22	286.25 ± 5.32	106.25 ± 4.16
Sham (9)	261.25 ± 2.95	220 ± 5.01****	306.25 ± 19.45****
Vehicle (8)	262.5 ± 2.67	219.75 ± 5.14****	333.75 ± 30.11****
DFE (8)	266.25 ± 3.75	247.25 ± 3.85^∗∗††^	288.87 ± 24.17****

Mean ± SEM (*n* = 8).

****P < 0.0001 versus control, **P < 0.01 versus control,  ^††^ *P* < 0.01 versus sham.

**Table 2:** The effect of DFE on histomorphometric parameters of rat sciatic nerve.

Groups	*N*	MMFD (*μ*m)	AD (*μ*m)	MSD (*μ*m)
Control	4	9.75 ± 0.45	5.11 ± 0.34	4.64 ± 0.35
DFE	5	9.61 ± 0.45	4.96 ± 0.25	4.65 ± 0.34
Sham	4	8.15 ± 0.19*	4.09 ± 0.15*	4.06 ± 0.23

*N*: number of animals; MMFD: Mean-Myelinated Fiber Diameter; AD: Axon Diameter, MSD: Myelin Sheath Diameter; Data are presented as Mean ± SEM. *P < 0.05 versus control.
